# Rare External Jugular Vein Pseudoaneurysm

**DOI:** 10.5811/cpcem.2019.12.45076

**Published:** 2020-03-02

**Authors:** Patrick J. Wallace, Jordana Haber

**Affiliations:** University of Nevada Las Vegas, Department of Emergency Medicine, Las Vegas, Nevada

## Abstract

External jugular vein pseudoaneurysm is a very rare cause of a neck mass due to the low pressure venous system. This case demonstrates a 27-year-old female who presented to the emergency department with a non-tender, compressible, left-sided neck mass that enlarged with valsalva and talking, and intermittent paresthesias. Upon workup, she was diagnosed with an external jugular vein pseudoaneurysm. Complications of this diagnosis are mentioned in the literature; however, most patients with an external jugular vein pseudoaneurysm or aneurysm can be safely discharged with close follow-up with a vascular surgeon.

## INTRODUCTION

Venous aneurysms are very rare compared to arterial aneurysms.^1^–^7^ This is postulated to be related to the low pressure system in the superior vena cava.^2^,^3^,^6^,^8^ Because of this 77% of venous aneurysms are located in lower extremities.^7^ In addition, pseudoaneurysms of the external jugular vein are less common than aneurysms of the internal jugular vein, making them an exceedingly rare entity.^3^,^5^,^7^

An aneurysm is defined as a dilation of all three layers of the vein wall. The histology of an aneurysm may show thinning of the elastic fiber wall, decreased smooth muscle in the media, and replacement of smooth muscle by fibrous tissue,^7^,^9^,^10^ whereas pseudoaneurysm is a tear through the outer layers of venous wall, the tunica adventitia and tunica media. The histology of a pseudoaneurysm shows collection of blood and thrombus in the wall.

## CASE REPORT

An otherwise healthy 27-year-old African-American female presented to the emergency department complaining of a left neck mass with associated paresthesias radiating up her left lateral neck and down the left arm. These paresthesias were intermittent and positional. She noticed the mass present suddenly about two months prior to presentation and endorsed gradual increase in size, in addition to intermittent and positional paresthesias. The mass was painless and enlarged with talking and valsalva maneuvers. She denied any recent interventions including massage, chiropractics, or neck manipulation. She had no known personal or family history of connective tissue disease. The patient also denied social history including smoking, alcohol, or substance use. However, she had been in a motor vehicle accident about four months prior without any significant injuries or immediate complications. This was a low-speed crash and the patient was able to self-extricate and self-ambulate without assistance immediately after the injury. No medical evaluation or imaging was completed at that time.

**Image 1:**
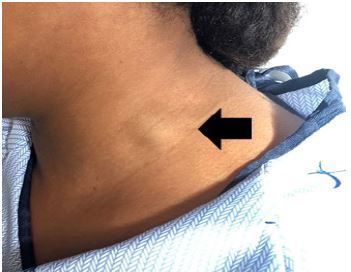
Two-centimeter left external jugular pseudoaneurysm as seen on physical exam.

On physical exam, she had a 2-centimeter (cm) soft, compressible, non-pulsatile, nontender mass that enlarged with valsalva and talking along the antero-lateral left neck (Image 1). The mass was soft and mobile. It did not move or change in size with respirations or swallowing. There was no overlying erythema, warmth, ecchymosis, induration, or surrounding lymphadenopathy. Strength and sensation were intact in all extremities. Adson’s, Allen’s, and Roo’s tests were all normal. Her paresthesias were not exacerbated with movement of her neck or arm, compression or distraction of the neck, or with Spurling’s maneuver. Her neck was non-tender without restriction of motion, hypertonicity of muscles, or edema.

**Image 2:**
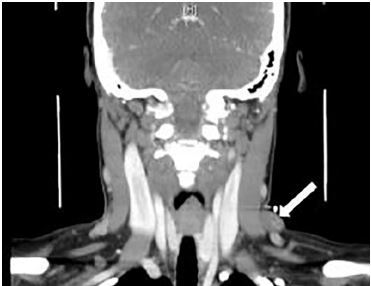
Computed tomography with contrast coronal view of left external jugular venous pseudoaneurysm (arrow).

**Image 3:**
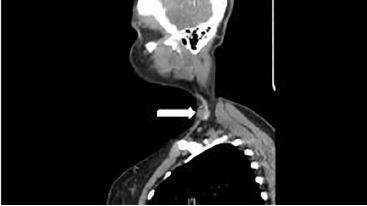
Computed tomography with contrast sagittal view of left external jugular venous pseudoaneurysm (arrow).

Basic blood work showed no abnormal findings. Computed tomography (CT) of the neck with contrast showed a 1.7 cm × 1.4 cm × 2 cm pseudoaneurysm of the left external jugular vein (Images 2 and 3). The case was discussed with the vascular surgeon on call. The patient had no signs that the pseudoaneurysm was expanding, causing airway compromise, had active extravasation, or was causing emergent neurological involvement at that time. We agreed she was safe for discharge at that time and could follow up with vascular surgery as an outpatient.

## DISCUSSION

We found minimal literature on the topic of external jugular vein aneurysm and pseudoaneurysm, with only two other case reports of pseudoaneurysm published. We could not find any articles that had performed a formal review of the literature. The table below compares the case reports available in this field of research.

**CPC-EM Capsule**

What do we already know about this clinical entity?

External jugular venous pseudoaneurysm is a rare presentation. It is rarely symptomatic and often caused by trauma or cannulation of the internal jugular vein.

What makes this presentation of disease reportable?

Although ultrasound or computed tomography (CT) with angiography is recommended, in this case we demonstrate CT with contrast is sufficient to make the diagnosis.

What is the major learning point?

Ultrasound is the standard of care imaging modality. If asymptomatic, most pseudoaneurysms can be safely discharged and follow up with vascular surgery as an outpatient.

How might this improve emergency medicine practice?

Complications are very rare and often not life-threatening and can be managed as an outpatient.

The most common presentation for aneurysm and pseudoaneurysm is a pulsatile, palpable mass that enlarges with valsalva.^1^,^10^ Other symptoms include pain, dysphagia, hoarseness, and neurological findings.^1^,^6^ Doppler ultrasound is the gold standard and recommended first imaging technique for aneurysms and pseudoaneurysms.^5^,^11^

Ultrasound can show turbulent flow and dilation with 95% accuracy for pseudoaneurysm.^1^ It is non-invasive and helps differentiate vascular from non-vascular causes. Arterial pseudoaneurysms are seen as pulsatile and turbulent waveform on Doppler ultrasound.^4^ (CT angiography, magnetic resonance imaging, and magnetic resonance venography can more accurately demonstrate size and extent, but are not first line.^5^,^6^,^8^,^11^,^12^ Additionally, CT with intravenous contrast may be a suitable imaging modality in cases where ultrasound or clinical uncertainty requires a CT without angiography. To our knowledge this is the only case in the literature where an external jugular vein pseudoaneurysm was diagnosed with a contrast CT without angiography.

Venous aneurysms are classified as primary (congenital) and secondary (acquired).^2^,^5^,^8^ Causes of primary venous aneurysms are not fully understood,^9^,^13^ while possible etiologies for secondary aneurysms within the venous system include thoracic outlet obstruction, trauma, chronic inflammation, degeneration, and increased venous pressure.^2^,^4^,^5^,^11^,^14^ Known risk factors for secondary venous aneurysms include recent trauma, cardiovascular disease, and age.^3^

Pseudoaneurysms in the arterial system of the neck have similar underlying etiology to include trauma,^1^,^3^ venous valve insufficiency,^3^ tumor,^3^ and iatrogenic causes such as surgical interventions or central line complications.^1^ There are currently only two other case reports on external jugular vein pseudoaneurysm.^5^,^12^ Shah et al discusses one of these case reports, specifically a fusiform dilation. This patient had no past medical history and no evidence of trauma other than repeated irritation to the neck by the sling and buckle of his rifle.^12^

Venous aneurysms and pseudoaneurysms are a rare cause of neck masses.^4^,^15^ The differential diagnosis includes lymphocele, cavernous hemangioma, hygroma, abscess, cyst, laryngocele, lymph node, tumor, thyroglossal cyst, and branchial cleft cyst.^4^,^5^,^12^,^15^ Enlargement of the mass with valsalva or excursion is suspicious for laryngocele, aneurysm, or pseudoaneurysm.^8^,^10^

Complications may include pulmonary embolism, thrombus formation or thrombophlebitis, and rupture.^5^,^6^,^8^,^10^,^12^ The research suggests there is risk of major embolic complications from jugular vein aneurysms. However, McCready et al states: “Based on the few cases in the literature, rupture or thromboembolic complications in patients with axillary or subclavian venous aneurysms do not appear to occur. Conservative therapy is appropriate for patients with axillary and subclavian venous aneurysms.”^10^ At the time of this publication there were no reports in the literature of any of the above-mentioned complications from external jugular vein pseudoaneurysm. These complications are mostly seen in the lower extremities from popliteal and femoral aneurysms.^7^,^10^,^14^

Surgical indications include large aneurysms compressing nearby structures, potential for thrombus, cosmetic reasons, or presence of symptoms.^7^,^10^ Venous aneurysms of the neck are often asymptomatic requiring no intervention and can be monitored.^1^,^2^,^5^ Approximately 89% of iatrogenic pseudoaneurysms will heal spontaneously without intervention.^1^ Upon our review, the case reports where patients underwent surgery were all for cosmetic reasons. Management can include supportive care and outpatient follow-up with surgery. Symptomatic patients may need to be admitted for observation if there are concerns for rapidly enlarging pseudoaneurysm, rupture, or signs of hemodynamic instability. As noted in the table below, none of the patients discussed in these case reports had concerning symptoms that would warrant admission and we believe the majority of patients with jugular vein pseudoaneurysms can be safely discharged and follow-up with a vascular surgeon.

## CONCLUSION

Pseudoaneurysms of the external jugular vein are very rare with only two other case reports published in the literature. Pseudoaneurysm presents as a pulseless mass that enlarges with valsalva and exertion. No complications have been reported in the literature and no intervention is indicated in the asymptomatic patient. If asymptomatic, patients can be safely discharged with outpatient referral to surgery for cosmetic excision.

## Figures and Tables

**Table:** Comparison list of articles on the topic of aneurysm and pseudoaneurysm of the external jugular vein.

Author, date	Age	Location	Type	Symptomatic (excluding swelling)	Intervention	Complications	Size	Medical History
Ekim et al. 2002^9^	21 M	External Jugular	Aneurysm	No	Surgical	None	2.0 cm diameter	None
Mohanty et al. 2013^11^	12 M	External Jugular	Aneurysm	No	Surgical	None	3.4 × 3.3 × 3.0 cm	None
Lucatelli et al. 2017^15^	56 F	External Jugular	Aneurysm	No	Surgical	None	3.0 × 3.0 cm	Hypertension, mixed connective tissue disease
Lee et al. 2006^7^	63 F	External Jugular	Aneurysm	No	None	None	2.8 cm diameter	None
Kirmani et al. 2011^8^	35 M	External Jugular	Aneurysm	No	None	None	3.0 × 1.5 cm	None
Karapolat et al. 2004^6^	4 M	External Jugular	Aneurysm	No	Surgical	None	1) 1.0 × 1.0 cm; 2) 2.0 × 1.0 cm	none
Grigorescu et al. 2012^5^	58 F	External Jugular	Pseudoaneurysm	Yes. Cervical constriction, pulsatile burning sensation	Avoid excessive physical exertion	None	3.5 × 3.0 cm	Hypertension, atrial fibrillation, mitral and aortic regurgitation, pulmonary hypertension
Drakonaki et al. 2011^4^	74 F	External Jugular	Aneurysm	No	None	None	2.2 cm	Internal jugular vein catheterization 2 years prior
Chapman et al. 2018^3^	75 F	External Jugular	Aneurysm	No	Surgical	None	“Ping pong-ball size”	Hypertension
Basbug et al. 2015^2^	19 F	External Jugular	Aneurysm	No	Surgical	None	2.5 × 3.5 × 1.5 cm	Lipoma excision Right supraclavicular region 11 years prior
Regina et al. 1992^14^	39 F	External Jugular	Aneurysm	No	Surgical	None	4.0 cm	None
Shah et al. 2015^12^	25 M	External Jugular	Pseudoaneurysm	No	Surgical	None; of note hemorrhage and thrombosis were present on histology	7.0 × 6.0 cm	None
Swaika et al. 2013^13^	8 M	External Jugular	Aneurysm	No	Surgical	None	2.0 × 1.0 cm	None

*M*, male; *F*, female; *cm*, centimeter.
